# Regular Medications Administered to Older Adults in Aged Care Facilities: A Retrospective Descriptive Study

**DOI:** 10.1111/jocn.17856

**Published:** 2025-06-16

**Authors:** Stephanie M. Garratt, Maneesh Prasad, Kelly Ottosen, Elizabeth Manias

**Affiliations:** ^1^ School of Nursing and Midwifery Monash University Melbourne Victoria Australia

**Keywords:** long‐term care, medication administration, medication therapy management, nursing homes, older people, residential aged care

## Abstract

**Aim(s):**

To explore which regularly prescribed medications are most commonly administered to older adults in aged care facilities in Australia, by whom and when, and to identify the prevalence of polypharmacy in this population group.

**Design:**

Retrospective descriptive study.

**Methods:**

This study involved exploratory analysis of de‐identified medication administration records from March 17, 2023–March 18, 2024. Older adults' demographic and medication administration data were requested from two electronic medication chart providers in Australia. For inclusion, older adults must have been living in an aged care facility for the entire timeframe. Data were analysed using descriptive statistics, activity pattern analysis, Welch two sample t‐tests, ANOVA and independent sample t‐tests. The STROBE checklist was used to report this study.

**Results:**

In all, 12,438 older adults were included, with a median age of 87, spanning 287 aged care facilities across Australia. Nervous system medications (over 16 million doses) and alimentary tract/metabolism medications (over 12 million doses) were the most administered. Within these, paracetamol 500 mg tablets and docusate sodium 50 mg + sennoside B 8 mg tablets were the most common. Quetiapine, a strong anticholinergic medication, was also present in the top 30 most administered medications. Certified nursing staff were the primary administrators of medication (66% of actions), followed by non‐nursing staff (27%). Medications were predominantly administered before 10 am and after 10 pm. With a median of 8 regular medications administered per older adult per day, 78% experienced polypharmacy.

**Conclusion:**

The most common regular medications administered in aged care facilities were non‐opioid analgesics and laxatives. Many medications were administered in the late evening, where staffing levels were likely to be limited. There was a high prevalence of polypharmacy, and non‐nursing staff were involved in medication administration.

**Implications for the Profession and/or Patient Care:**

This study offers important insights and new knowledge around use of regular medications in aged care facilities, using a nationally representative sample from Australia. It highlights the high volume of non‐opioid analgesics and laxatives administered to older adults, some of which may be optimised, modified or replaced with nonpharmacological alternatives to reduce medication burden. This study also notes that not all regular medications are being administered in Australia by certified nursing staff, and that medication administration activity peaks during both breakfast and late evening rounds. These are important considerations for aged care facilities when assessing staffing ratios, rostering, and how to reduce competing demands for aged care staff. Although much attention has been placed on reducing polypharmacy and optimising medications for older adults, this study also identifies that polypharmacy is prevalent, with 78% of older adults experiencing this through use of regular medications alone. The findings of this study will enable more informed discussions between nursing staff, prescribers, pharmacy and potentially older adults and their families around regular medication and its administration in aged care facilities.

**Reporting Method:**

The STROBE checklist was followed.

**Patient or Public Contribution:**

No patient or public contribution.

**Summary**

- What problem did the study address?
  ○ Insights around medication administration in aged care facilities typically stem from dispensing/prescribing data, audits across multiple sites governed by one organisation, or small sample sizes. However, not all medications that are dispensed or prescribed are administered to older adults in care. This study demonstrates that it is possible to achieve population‐level insights into which regular medications are truly administered in aged care facilities through the use of administration data from electronic national residential medication charts.
- What were the main findings?
  ○ The top 30 most administered medications included high volumes of non‐opioid analgesics and laxatives, with psycholeptic medications such as quetiapine and risperidone also featuring. Over 25% of regular medications were administered by non‐nursing staff. Seventy‐eight percent of older adults experienced polypharmacy (five or more daily unique medications) through their regular medications. Number of rounded mean number of regular daily medications was not associated with older adults' age or gender.
- Where and on whom will the research have an impact?
  ○ This study may serve as a foundation for deprescribing, quality use of medicines and nonpharmacological initiatives in aged care facilities that seek to reduce medication burden on older adults. Findings may also be of interest to older adults and their families, as a starting point for conversations and shared decision‐making about regular medications.
- What does this paper contribute to the wider global clinical community?
  ○ This study is the first known to investigate the administration of regular medications in Australian aged care facilities on a national level, representing all states and territories. It found that the most commonly administered medications were analgesics (paracetamol 500 mg tablets; paracetamol 665 mg tablets modified release) and laxatives (docusate sodium 50 mg + sennoside b 8 mg tablets; macrogol‐3350 powder for oral liquid).
  ○ Psycholeptic medications were present in the top 30 most administered medications, with quetiapine 25 mg tablets (23rd) and risperidone 500 μg tablets (25th) represented, in addition to mirtazapine 15 mg tablets (19th), a psychoanaleptic medication.
  ○ Polypharmacy remains an ongoing concern in aged care facilities. This study found that 78% of older adults received over five unique medications daily as regular medication.

## 1 Introduction

Aged care facilities, known elsewhere as nursing homes or long‐term care facilities, provide supported accommodation and 24/7 nursing care for older adults with care needs who can no longer live independently in their own homes (Sluggett et al. 2017). Older adults living in aged care facilities are more likely to be frail, medically complex and have high levels of medication use (Cross et al. 2024; Page et al. 2024).

Medication administration is a key clinical service carried out by aged care staff who manage and administer medications to older adults, ensuring that the right medication, in the right dosage, reaches the right person at the right time via the right route, with the right documentation filled out (Grissinger 2010). Medication administration requires a high level of clinical and interpersonal skill and can be a time‐intensive activity—particularly during morning medication administration (Garratt et al. 2024). In Australia, as with other jurisdictions, there are guiding principles and standards set out on a national level that are designed to guide aged care staff around safe medication management and administration (Cross et al. 2024; Department of Health and Aged Care 2022). Yet, although older adults may no longer be managing and administering their own medications as what happens in private home settings, there is still a risk of medication error if a mistake is made by aged care staff.

Medication administration errors have both been widely researched internationally; they are known to increase older adults' risk of medication‐related harm and adverse events (Garratt et al. 2024). In their study of medication administration errors and the incidence of adverse events, Al‐Jumaili and Doucette (2018) found a rate of 6.13 adverse events per 100 older adults in aged care facilities per month, with significant associations between adverse events and older adults who received psychotropic medications, opioids or warfarin. Similarly, in Australia, the most common medication classes referred to in complaints data include opioids, psychotropic medication and insulin (Breen et al. 2023). In response to concerns around high medication use and polypharmacy in aged care facilities internationally, quality care initiatives, research and policy have primarily focussed on deprescribing, polypharmacy or medication regimen optimisation for older adults in aged care facilities, reducing the administration of unnecessary medication (Page et al. 2023; Poudel et al. 2024).

Electronic medication records have also been introduced, particularly in Australia and New Zealand, reducing medication errors and increasing aged care staff's visibility of medication records compared to paper‐based records (Elliott et al. 2016; Lei et al. 2023). Pilot studies in Australia identified that electronic National Residential Medication Charts (eNRMC products) facilitate faster and more accurate medication reviews through easier access to longitudinal administration data (Elliott et al. 2016; McDerby et al. 2019).

Previous studies that investigate quality use of medicines in aged care facilities have used prescription or dispensing data through national databases (Kalisch Ellett et al. 2019). However, not all prescribed or dispensed medication doses may actually be administered to older adults in aged care facilities, as a result of medication omissions (e.g., delays in delivery from the pharmacy, hospitalisation of an older adults or medication refusal) (Garratt et al. 2024).

In addition, studies which focus on the prevalence of specific medication classes or types in aged care facilities, such as anticholinergic medication (Cross et al. 2024), psychotropic medication (Harris and Lykina 2022), analgesics (La Frenais et al. 2021) or Pro Re Nata, or as required medications, (Sharma et al. 2021) typically involve repeated audits or medication reviews carried out by pharmacists for a small number of aged care facilities (Poudel et al. 2024). These studies are limited in terms of generalisability, through their small sample sizes (e.g., using one aged care provider or organisation). By focussing on specific medication classes/types, provider or site‐specific audits, or prescribing/dispensing data, prior studies have attempted to overcome limitations such as volunteer bias, manual conduct of audits and small cross‐sectional samples.

## 2 Aim

With the rapid expansion of eNRMC products in Australia and internationally, it is possible to conduct investigations into national administration trends, pooling administration data from eNRMC providers. By using eNRMC administration data, a more accurate picture of what medications are actually administered to older adults in aged care facilities is achievable, rather than relying on records of what has been prescribed or dispensed, where not all doses may be administered.

This study aimed to explore regularly prescribed medications administered to older adults in aged care facilities in Australia, using medication administration data. The objectives of this study were to identify: (1) the most commonly administered regular medication; (2) who administered regular medication in Australian aged care facilities; (3) when regular medications were administered and (4) the prevalence of polypharmacy in this population group.

## 3 Methods

### 3.1 Study Design and Setting

This retrospective descriptive study was exploratory in nature, conducted using a sample of routinely collected medication administration data from Australian aged care facilities. The sample was designed to span one calendar year, from March 17, 2023 to March 18, 2024, and was sourced from two electronic National Residential Medical Chart (eNRMC) providers. This study adhered to the Strengthening the Reporting of Observational Studies in Epidemiology (STROBE) guidelines (Appendix S1).

### 3.2 Study Setting and Sampling

The sample for this study was drawn from two eNRMC providers, who when combined, were operational across all states and territories of Australia, with their software used in over 400 aged care facilities. Records from aged care facilities using each eNRMC product were eligible for inclusion if they had used the product for the entire timeframe, from March 17, 2023 to March 18, 2024. Older adults living in these aged care facilities were eligible for inclusion in the sample if they had resided in care at an included aged care facility for the whole timeframe, as the eNRMC providers are not able to track specifically why older adults might enter and exit an aged care facility (e.g., for a short stay/respite care, moving facilities, or passing away). Older adults were also required to have had regular medications managed using eNRMC, administered to them by aged care staff. The sample size for older adults and aged care facilities was not pre‐determined, due to the exploratory nature of this study. The sample included demographic details such as age of older adults and their sex, in addition to name of medication, date/time of medication administration and role of the staff member that administered the medication.

Only regular prescribed medications that were administered by aged care staff were requested in the data samples for this study. Pro Re Nata (PRN) and short course medications were excluded as they are potential confounding factors for any longitudinal analysis of medication administration and should be considered separately. These medication types are subject to additional influences and clinical decision‐making; they are intended for short‐term use (e.g., short course antibiotics for an infection) or for sporadic use in response to acute need (e.g., PRN pain medication for breakthrough pain) (Kalisch Ellett et al. 2019; Sharma et al. 2021).

### 3.3 Data Cleaning

All identifiers for aged care facilities, older adults and aged care staff were removed and replaced by numerical identifiers by the eNRMC providers prior to data being made available to the research team. Data were managed and cleaned using R (v 4.4.1). In total, 2449 rows (0.006%) from eNRMC provider one and 6598 rows (0.002%) from eNRMC provider two were removed during the cleaning process, due to parsing issues. Staff role permissions were cleaned into two categories, ‘certified nursing’ (e.g., registered nurses, enrolled nurses) and ‘non‐nursing’ staff (e.g., care assistants).

Medication‐related data were included in a variety of ways, using brand name, generic name or a combination of both depending on what had been entered by prescribers into eNRMCs. Non‐standardised drug names, free text drug entries and spelling errors posed a significant data‐cleaning challenge. Specialised code in R was developed to match medicine names to Medicinal Product Unit of Use (MPUU) from the Australian Medicines Terminology (AMT), a subset of the Systematised Medical Nomenclature for Medicine–Clinical Terminology (SNOMED CT‐AU), downloaded from the National Clinical Terminology Service (Australian Digital Health Agency 2021, 2024). Gestalt pattern matching was used, a string‐matching algorithm for determining the similarity of two strings (medication names) and providing a score to show how alike any two strings (medication names) are. Matches below a similarity score of 0.9 were manually checked and adjusted by two members of the research team, including a pharmacist to ensure reliability and no loss of mismatched data. The sample also included a large number of over‐the‐counter products/complementary medicines (e.g., multivitamins; moisturisers) and non‐therapeutic agents. Similar to prior studies, over‐the‐counter products were categorised separately and excluded from analysis, as were regular reminders about care‐related activities which were noted in the medication lists (Page et al. 2023). This was because this study aimed to focus on conventional, regular prescribed medications.

Anatomical Therapeutic Chemical (ATC) codes were added to the dataset to allow for the classification and grouping of medications across five levels, from active ingredients (fifth level) through to anatomical system (first level). (World Health Organization 2024). ATC codes are internationally used to compare medication use, monitor adverse drug reactions, inform policy development and are a standardised method of classification for medications.

### 3.4 Data Analysis

This study undertook an exploratory analysis of secondary data. Data were summarised descriptively using a medication administration summary count, medians, means, standard deviations and frequencies. To assess the timing of administration for regular medications, an activity summary across a 24‐h period was generated using timestamps for each dose administration. Significance for statistical tests was set at *p* < 0.01, given the high number of individuals within the sample, in order to reduce the risk of Type I error. Inferential tests conducted to investigate potential relationships between older adults' gender, age and polypharmacy included Spearman's correlation coefficient, Tukey's HSD tests, Welch two‐sample *t*‐tests, and independent sample *t*‐tests. In keeping with prior studies on medication use in aged care facilities, polypharmacy was defined as five or more unique regular medications per day (Page et al. 2023).

### 3.5 Ethics Statement

Ethical approval was obtained from the Monash University Human Research Ethics Committee (ID: 41473). The data in this study, obtained from electronic medication administration records, had identifiable information removed before being provided to the research team.

## 4 Results

### 4.1 Sample Demographics

In total, data were received for 12,438 unique older adult identification numbers, representing those who had resided in care for the entire timeframe (March 17, 2023 to March 18, 2024), across 287 unique aged care facilities in Australia. This sample represents 11% of all aged care facilities in Australia operating as of June 30, 2024 (Australian Institute of Health and Welfare 2024). Between the two eNRMC providers, all states and territories of Australia were represented in the data. Demographic characteristics of older adults and aged care facilities are summarised in Table 1. Sixty‐eight percent of older adults were identified as female, and the median age was 87 years (IQR 81–92 years). A mean of 43 older adults per aged care facility were included in the sample, having been in care for the entire sample timeframe.

**TABLE 1:** Sample demographics (*n* = 12,438 older adults).

Electronic national residential medication chart (eNRMC), *n* of older adults (%)	
Provider 1	8529 (69%)
Provider 2	3909 (31%)
Sex, *n* (%)	
Male	3814 (31%)
Female	8434 (68%)
Unknown	190 (1%)
Age (years)	
Median (IQR)	87 (81–92)
Number of residents per aged care facility over timeframe	
Mean (SD.)	43 (26)

### 4.2 Commonly Administered Regular Medications

A wide range of regular medications were administered in aged care facilities in Australia, with the top 30 most administered medications summarised in Table 2. Over the timeframe, paracetamol 500 mg tablets (*n* = 4,946,906) were the most commonly administered medication, linked to 48% of unique older adult IDs. Paracetamol 665 mg modified release tablets (1,660,633 doses across 16% of older adults) were also in the top five most administered medications, ranking third. Other medications in the top five included two laxatives, docusate sodium 50 mg + sennoside b 8 mg tablets (ranked 2nd, 2,056,769 doses across 55% of older adults) and macrogol‐3350 13.125 g + sodium chloride 350.7 mg + sodium bicarbonate 178.5 mg + potassium chloride 46.6 mg powder for oral liquid (ranked fifth, 1,202,816 doses, 27% of older adults). Cholecalciferol 25 μg capsules were the fourth most administered medication (1,497,939 doses, 20% of older adults).

**TABLE 2:** Audit of regular medications by count (top 30 presented).

Rank	Medication name	Anatomic therapeutic chemical code	Proportion of unique older adult IDs (%)	Doses dispensed (*n*)	Doses administered (*n*)	Proportion administered (%)
1	Paracetamol 500 mg tablet	N02BE01	48%	5,052,200	4,946,906	97.9%
2	Docusate sodium 50 mg + sennoside B 8 mg tablet	A06AB56	55%	2,118,409	2,056,769	97.1%
3	Paracetamol 665 mg modified release tablet	N02BE01	16%	1,693,413	1,660,633	98.1%
4	Colecalciferol 25 μg (1000 units) capsule	A11CC05	20%	1,513,606	1,497,939	99.0%
5	Macrogol‐3350 13.125 g + sodium chloride 350.7 mg + sodium bicarbonate 178.5 mg + potassium chloride 46.6 mg powder for oral liquid, sachet	A06AD15	27%	1,307,634	1,202,816	92.0%
6	Macrogol‐400 0.4% + propylene glycol 0.3% eye drops	S01XA20	11%	1,078,660	1,048,259	97.2%
7	Metoprolol tartrate 50 mg tablet	C07AB02	12%	960,174	948,266	98.8%
8	Cholecalciferol 25 μg (1000 units) tablet	A11CC05	37%	824,695	816,349	99.0%
9	Furosemide 20 mg tablet	C03CA01	19%	748,933	737,811	98.5%
10	Pantoprazole 40 mg enteric tablet	A02BC02	17%	707,898	700,072	98.9%
11	Apixaban 2.5 mg tablet	B01AF02	9%	679,884	670,655	98.6%
12	Aspirin 100 mg tablet	B01AC06	17%	649,335	640,239	98.6%
13	Furosemide 40 mg tablet	C03CA01	40%	645,108	626,207	97.1%
14	Apixaban 5 mg tablet	B01AF02	7%	494,918	488,998	98.8%
15	Pantoprazole 20 mg enteric tablet	A02BC02	10%	374,073	370,268	99.0%
16	Levodopa 100 mg + carbidopa 25 mg tablet	N04BA02	3%	362,294	357,773	98.8%
17	Esomeprazole 20 mg enteric tablet	A02BD06	9%	350,907	346,821	98.8%
18	Dextran‐70 0.1% + hypromellose 0.3% eye drops	S01XA20	3%	349,130	338,975	97.1%
19	Mirtazapine 15 mg tablet	N06AX11	10%	337,923	332,814	98.5%
20	Pregabalin 25 mg capsule	N02BF02	7%	332,187	328,761	99.0%
21	Melatonin 2 mg modified release tablet	N05CM17/N05CH01	10%	328,364	322,860	98.3%
22	Spironolactone 25 mg tablet	C03DA01	9%	326,099	321,846	98.7%
23	Quetiapine 25 mg tablet	N05AH04	6%	316,156	309,674	98.0%
24	Calcium carbonate 1.5 g (calcium 600 mg) tablet	A12AA04	11%	312,928	309,175	98.8%
25	Risperidone 500 μg tablet	N05AX08	6%	312,659	307,018	98.20%
26	Calcium carbonate 1.5 g (calcium 600 mg) + colecalciferol 12.5 μg (500 units) tablet	A12AX	6%	298,051	294,169	98.70%
27	Clopidogrel 75 mg tablet	B01AC04	7%	296,393	292,749	98.8%
28	Amlodipine 5 mg tablet	C08CA01	8%	295,859	292,384	98.8%
29	Hyaluronate sodium 0.2% eye drops	S01XA20	3%	300,057	291,141	97.0%
30	Metformin hydrochloride 500 mg tablet	A10BA02	5%	278,285	275,342	98.9%

The top 30 most administered medications also included two atypical antipsychotics. Risperidone 500 μg tablets were ranked the 25th most administered medication (307,018 doses over the timeframe), across 6% of older adults; and quetiapine 25 mg tablets were ranked the 23rd most administered regular medication (309,674 doses), 6% of unique older adult IDs (Table 2). In addition, an atypical antidepressant, mirtazapine 15 mg tablets were administered to 10% of all unique older adults (19th most administered regular medication, 332,814 doses).

When grouped by ATC classifications (first level, anatomical system), nervous system medications were the most administered over the time frame, with over 16 million doses (Table 3). The next most administered were alimentary tract and metabolism medications, with over 12 million doses (Table 3). Of the 14 ATC codes, only one classification, antiparasitic products, had an administered proportion less than 95%, indicating a higher volume of medication omissions by aged care staff compared to other ATC codes.

**TABLE 3:** Administered anatomical therapeutic chemical (ATC) classifications (first level), regular medications.

Anatomical therapeutic chemical classification (1st level)	Description	Doses dispensed (*n*)	Administered doses (*n*)	Proportion administered (%)
A	Alimentary tract and metabolism	12,531,452	12,200,242	97.4%
B	Blood and blood forming organs	2,949,508	2,906,002	98.5%
C	Cardiovascular system	7,855,333	7,747,561	98.6%
D	Dermatologics	1,234,688	1,181,646	95.7%
G	Genito urinary system and sex hormones	426,857	413,340	96.8%
H	Systemic hormonal preparations (excludes sex hormones and insulins)	954,958	941,399	98.6%
J	Anti‐infective for systemic use	941,119	901,078	95.8%
L	Antineoplastic and immunomodulating agents	138,485	134,421	97.1%
M	Musculoskeletal system	843,116	819,499	97.2%
N	Nervous system	16,341,813	16,042,703	98.2%
P	Antiparasitic products	2811	2389	85.0%
R	Respiratory system	1,634,473	1,583,439	97.0%
S	Sensory organs	3,776,120	3,643,838	97.0%
V	Various	36,103	34,765	96.3%

### 4.3 Administrators of Regular Medications

Certified nursing staff, registered or enrolled nurses, were the primary administrators of regular medication in aged care facilities across the sample (66% of all administration actions recorded), followed by non‐nursing staff, identified as personal care assistants (27%).

### 4.4 Timing of Regular Medication Administrations

**FIGURE 1:**
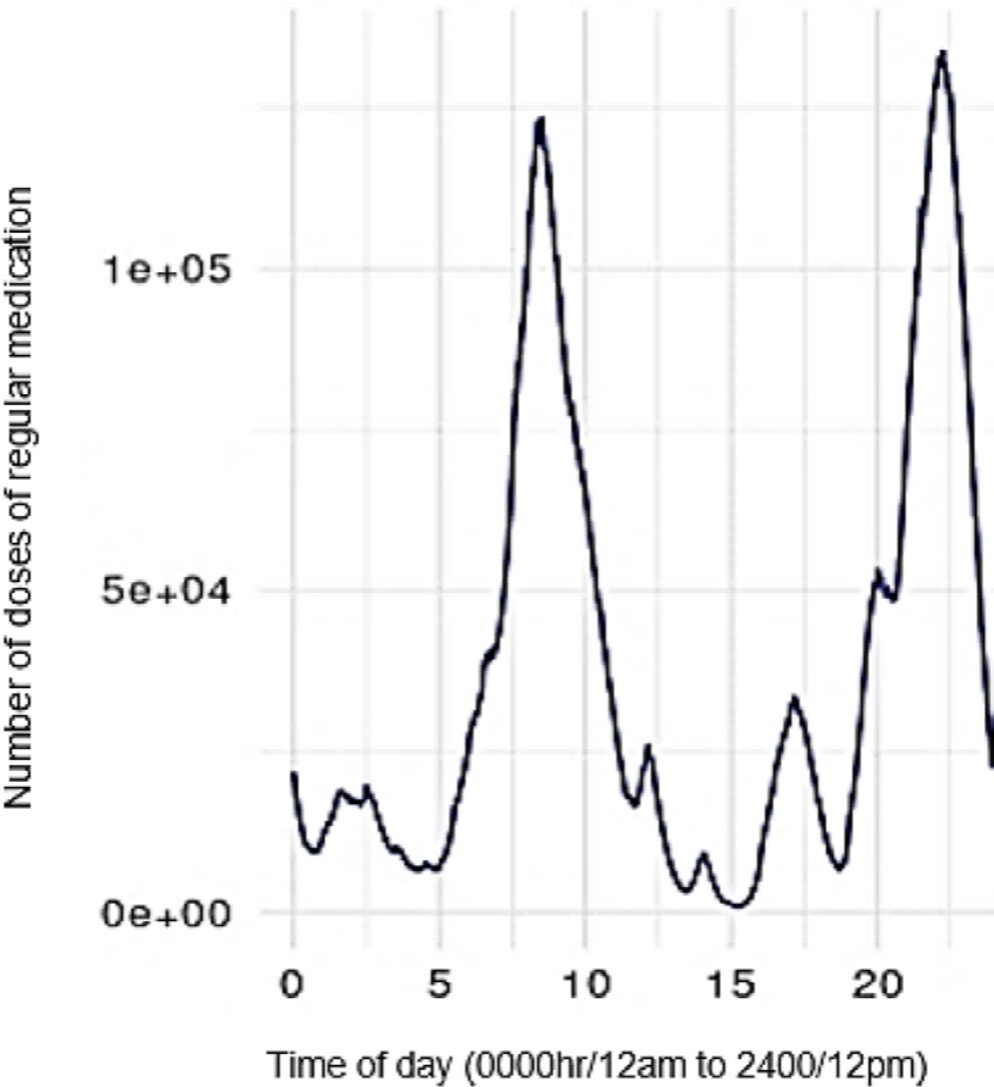
Daily activity summary for regular medication administration actions. [Colour figure can be viewed at wileyonlinelibrary.com]

Activity levels for administration of regular medications are detailed in Figure 1, with time in hours beginning at 0 (0000 h/12 am) and ending at 24 (2400 h/12 pm). There were two ‘peaks’, one situated over the timeframe for morning medication administration rounds (0700 h to 0900 h) and evening/bedtime rounds (2200 h/10 pm). The top 10 most common medications given between 1800 h (6 pm) and 2400 h (12 pm) include paracetamol 500 mg tablets, cholecalciferol 25 μg capsules/tablets, various forms of laxatives, pantoprazole 40 mg, aspirin 100 mg tablets, metoprolol 50 mg tablets and paracetamol 665 mg modified release tablets.

### 4.5 Prevalence of Polypharmacy

To conduct the polypharmacy analysis, the rounded mean number of unique regular medications per day per older adult was calculated. Overall, 78% of older adults were administered five or more unique regular medications per day. The median number of daily regular medications was calculated for each Australian state and territory represented in the data and is summarised in Table 4. Older adults received a median of 8 distinct regular medications per day (IQR 5–11) (Table 4). There was no significant relationship between older adults' gender and polypharmacy (Independent samples *t*‐test, *t* (8689) = −2.3, *p* = 0.02). Similarly, an ANOVA was conducted to investigate any association between state/territory and polypharmacy. The difference between means was significant (*F* (9) = 201,725, *p* < 0.01), despite the negligible practicable difference (Table 4).

**TABLE 4:** Median number of daily administered regular medications per resident, per state.

State	Median (IQR)
Australian Capital Territory	8 (5–10)
New South Wales	8 (5–11)
Northern Territory	7 (5–10)^a^
Queensland	8 (5–12)
South Australia	7 (5–10)
Tasmania	8 (5–10)^a^
Victoria	8 (6–12)
Western Australia	8 (6–13)^b^
Total Australia	8 (5–11)

^a^ eNRMC provider 2 was not operational in any aged care facilities in Tasmania or Northern Territory for the full timeframe.

^b^ eNRMC provider 1 was not operational in any aged care facilities in Western Australia for the full timeframe.

A very weak association was identified between older adults' age and rounded mean number of unique regular medications per day, with Spearman's correlation coefficient giving a *r*_s_ of −0.07 (*p* < 0.01). Using the youngest age group (years 65–74) as the reference, a Tukey's HSD test was performed, finding no statistically significant difference in rounded mean number of daily unique regular medications between age groups (Table 5). The oldest group, ages 105–114, was comprised of a very small sample, and the significant association for this group should be considered with caution.

**TABLE 5:** Tukey's HSD test between mean number of daily regular medications and age group.

Age group	Difference	Std Error	95% CI		*z*	*p*
			Lower	Upper		
65–74	Ref					
75–84	0.48	0.01	−0.03	0.04	0.39	0.69
85–94	1.93	0.01	−0.01	0.05	1.69	0.09
95–104	−2.93	0.01	−0.07	0.01	−2.09	0.04
105–114	−38.44	0.13	−0.71	−0.06	−3.09	0.00

## 5 Discussion

This is the first known study to investigate the administration of regular medications in Australian aged care facilities nationally, using medication administration records sourced from eNRMC providers. Nervous system medications were the most administered, particularly non‐opioid analgesics (paracetamol 500 mg tablets; paracetamol 665 mg tablets modified release), and alimentary tract and metabolism medications, (primarily docusate sodium 50 mg + sennoside B 8 mg tablets). Psycholeptic medications were also present in the top 30 most administered medications, with risperidone 500 μg tablets (25th), and quetiapine 25 mg tablets (23rd) represented, in addition to mirtazapine 15 mg tablets (19th), a psychoanaleptic medication. Not all regular medications were administered by qualified nursing staff, with over 25% of regular medications administered by non‐nursing staff who were not subject to the same regulatory processes or received the same level of training around medications. There were two peaks in major administration activity, the morning and late evening, which may have affected workload. Seventy‐eight per cent of older adults experienced polypharmacy over the timeframe, with a median of eight unique regular medications administered daily.

The study results comprised comprehensive information about the anatomical therapeutic chemical codes and specific medications within these codes. The focus of prior studies has been to report on specific medications or medication groups in regards to prescribing or dispensing practices, for example, analgesics (Sandvik et al. 2016) and psychotropic medications (Harris and Lykina 2022). This study showed that nervous system medications were the most commonly administered (over 16 million doses), and within this category, almost five million doses of paracetamol 500 mg tablets (across 48% of older adults) and 1.6 million doses of paracetamol 665 mg modified release tablets (across 16% of older adults) were administered to older adults across the study timeframe. This aligns with the international literature around analgesic use in older adults, where paracetamol is considered the first‐line analgesic for mild to moderate pain (Dowd et al. 2023), and is consistently reported as the most commonly prescribed, dispensed and administered analgesic for this population group (Mian et al. 2018; Sandvik et al. 2016). Dowd et al. (2023) found that 69% of older adults in their Australian sample of older adults across 12 aged care facilities were regularly charted paracetamol, and that one in three could benefit from an analgesic regimen review. There is a lack of evidence based on whether standardised dosing of paracetamol as a regular medication should be adapted for older adults, given the physiological changes that may occur as one ages (Mian et al. 2018; Sandvik et al. 2016). This is further complicated by challenges around accurate identification of pain and inappropriate prescribing of analgesics for older adults living with dementia (Sandvik et al. 2016). The current study and its identification of high volumes of paracetamol use reinforces Sandvik et al.'s (2016) recommendation that more targeted medication reviews be conducted to ensure appropriate use of paracetamol.

Alimentary tract and metabolism medications were highly administered (over 12 million doses). Two forms of laxatives featured in the top five most administered medications, administered to 55% and 27% of older adults. This is similar to Elli et al. (2021), who found 48% of older adults in their sample of 27 aged care facilities were receiving laxatives. Prior studies have identified tensions around the need for laxatives, and their association with prescribing cascades and use of particular medications, such as opioids and psychotropic medications in aged care facilities (Elli et al. 2021; Fosnes et al. 2012). Unfortunately, there is a lack of evidence around the efficacy of nonpharmacological interventions for constipation, such as mobilisation and adjusting diets (Dobarrio‐Sanz et al. 2020).

The current study found that quetiapine, considered a strong anticholinergic medication, was the 23rd most administered regular medication, with approximately 6% of the sample receiving this medication. Strong anticholinergic medication have been identified as potentially inappropriate for use in older adults, labelled medications to avoid in various prescribing criteria (American Geriatrics Society 2023). Quetiapine is often used as an off‐label medication, particularly for its sedative effect, despite international calls to avoid its use in older adults in general, with additional concerns around use in individuals with dementia, cognitive impairment, or delirium (American Geriatrics Society 2023; Cross et al. 2024; Garratt et al. 2020). However, reducing use of psycholeptic medication, and anticholinergic medication in general requires availability of alternative, appropriate management strategies (Breen et al. 2023), particularly nonpharmacological strategies.

The current study showed that 27% of regular medication doses were administered by non‐nursing staff, identified as personal care assistants. International discourse exists around the delegation of clinical care activities by registered nurses to unregulated workers, such as personal care assistants, known internationally as care assistants, unlicensed personnel, or personal care aids (Garratt et al. 2024; Oppert et al. 2018). In Australia, the guiding principles for medication management in aged care facilities indicate that medication administration may be delegated to personal care assistants. These individuals are not bound by professional practice standards and therefore they may not have undertaken vocational training around medications (Australian Government Department of Health and Aged Care 2023). Delegation of medication administration to unlicensed personnel such as personal care assistants has been met with unease from registered nurses and aged care managers. Although personal care assistants may be able to follow instructions on a medication chart or dose administration aid, because they may not have received adequate training on medications and best practice for administration, there is a possible increased risk of medication errors and complaints (Garratt et al. 2024; Gransjon Craftman et al. 2016; Tangiisuran et al. 2018).

Our study also showed that activity levels around medication administration peak at both breakfast and bedtime. Such increased activity levels are important for practice, as the ratio of aged care staff to residents is likely to be less in the late evening compared to early morning, despite the high activity levels around medication administration and personal care needs of residents. Prior time‐and‐motion studies typically focus on morning shifts, identifying that medication administration at breakfast is the most complex, laborious and time‐consuming (Chen et al. 2020; Qian et al. 2016). Conversely, Chen et al. (2020) also identified in their time‐and‐motion study that medication administration at bedtime required nearly as much time on average as breakfast. This may be due to medication, prescribing or resident‐related factors that determine the time medications are administered.

This study identified that 78% of older adults experienced polypharmacy across the sample. This is lower than prior Australian studies, which have found up to 92% of older adults may experience polypharmacy in aged care facilities (Page et al. 2023). Prior, smaller studies internationally have used similar inclusion criteria for their assessment of regular medications and polypharmacy, using medication chart reviews or dispensing/prescribing data which do not account for whether medications were administered or omitted (MacRae et al. 2021). Polypharmacy has been reported to increase the risk of adverse drug events (such as falls) and creates a high administrative burden for aged care staff, as well as a high medication burden for older adults (Page et al. 2023). Older adults living in aged care facilities are known to more commonly experience polypharmacy as a result of chronic conditions, prescribing cascades and complex comorbidities (MacRae et al. 2021). The current study, with its broader focus on regular medications over a longitudinal timeframe, found a statistically significant but very weak negative association between number of regular medications and age. This is in line with prior studies over the past 20 years that either report no association or a negative association between number of regular medications and age (Jokanovic et al. 2015). Although the relationship found by the current study is statistically significant, it is so small that it may have little to no practical application and is more of an indicator that older adults living in aged care facilities have a large number of unique regular medications administered daily, regardless of their age. High prevalence of polypharmacy in older adults living in aged care facilities is reported across studies and stronger associations with number of medications/polypharmacy have been found by prior studies with particular health conditions and comorbidities (Jokanovic et al. 2015; MacRae et al. 2021; Page et al. 2023). Given the clinical complexity of older adults in aged care facilities, a careful balance should be maintained between polypharmacy and under prescribing to achieve optimal, quality use of medications (Liau et al. 2024). Interventions to reduce polypharmacy should consider the views of older adults, their families and staff, informed by interdisciplinary medication reviews to ensure concordance and patient‐centred clinical decision‐making.

### 5.1 Limitations

This study has high generalisability due to its large sample size, longitudinal nature and inclusion of aged care facilities from all states and territories in Australia; it may not reflect aged care facilities with high levels of respite care and palliative care provision. This study excluded older adults who resided in aged care facilities temporarily, moved in during the timeframe, or passed away during the timeframe. It may be that the older adults represented in this sample had more stable health statuses. Older adults' health conditions and comorbidities were not included in this dataset, limiting our ability to comment on associations between polypharmacy and conditions such as dementia, which have previously been associated with higher experiences of polypharmacy in older adults living in aged care facilities (MacRae et al. 2021). Additionally, no indications for regular medications were included as part of the data request, and we were unable to analyse or discuss why medications had been prescribed more frequently than others.

Finally, the roles for aged care staff could not be separated into particular qualifications for analysis. Staff roles were classified regarding role permissions and were not consistently allocated across the eNRMC products. Each aged care facility selects the role/role permissions of each new staff member who is given access to their eNRMCs. The research team instead grouped role allocations into categories: those with nursing qualification level permissions and those who did not but were still given permissions to sign for and administer medication. It is recommended that a discussion be held on a policy and practice level with eNRMC providers in Australia to bring about a consistent labelling method of staff permissions/roles for audit and research purposes.

### 5.2 Future Research

The findings of this study indicate several avenues for future research into the complexity of medication administration in aged care facilities. First, there is an evidence gap around the utility and feasibility of nonpharmacological approaches to constipation, given the lack of methodological quality and risk of bias around randomised controlled trials in this area, and the continuing high use of laxatives in aged care facilities. Second, research is needed to better identify barriers to person‐centred strategies aimed at reducing the use of anticholinergic medications like quetiapine in aged care facilities (Rodrigues et al. 2024). Third, the benefits and challenges associated with delegating medication administration to personal care assistants requires assessment from a research, policy and practice level in Australia, particularly in light of education and quality care concerns, and limited guidance on a federal and state level. Finally, there is a need for further mixed‐methods research into medication administration and demands on aged care staff during evening and overnight shifts, given the limited evidence available, and similar medication administration activity to morning medication rounds.

## 6 Conclusion

A wide variety of medications are regularly administered to older adults in aged care facilities in Australia, with the most common medications including paracetamol and laxatives. Quetiapine, a strong anticholinergic medication, was also regularly administered to 6% of older adults. Over 75% of older adults experienced polypharmacy and given the complex and heterogeneous conditions of older adults in aged care facilities, it is associated with an increased risk of adverse events and high administrative burden for aged care staff. Interdisciplinary, collaborative medication reviews are needed that include the perspectives of older adults, their families and aged care staff, alongside prescriber and pharmacist perspectives. This collaborative approach may assist to further deprescribing initiatives and help to support quality, resident‐centric use of medications such as paracetamol, laxatives and psycholeptics in aged care facilities.

## 7 Relevance to Clinical Practice

High volumes of paracetamol and laxatives are regularly administered in aged care facilities. Better support is needed from policy and practice around using nonpharmacological approaches as first‐line treatment to manage conditions in aged care facilities and reduce medication burden for older adults. Our results show that quetiapine is commonly administered in aged care facilities, despite a clear lack of evidence to support its use in older adults, and risk of increased decline in cognitive and functional abilities (Garratt et al. 2020; Müller et al. 2020). Regular medication reviews, informed by older adults' conditions and particular medications should be conducted with input from older adults themselves and their family members where possible. Also, clarity is needed around what additional training non‐nursing certified staff undertake voluntarily in order to contribute to medication administration, given that they contribute to providing over a quarter of regular medication doses. Additional workforce support may be required in the late evening to support the likely increased workforce burden associated with medication administration. This study found that polypharmacy was common, but not all polypharmacy may be harmful, and prescribers should take care to distinguish between appropriate and inappropriate prescribing in aged care facilities, taking into account risk of adverse drug events (MacRae et al. 2021).

## Author Contributions

Conceptualisation of the study and funding acquisition was conducted by S.M.G., K.O. and E.M. Data curation, formal analysis and investigation was carried out by S.M.G. and M.P. S.M.G., K.O. and E.M. were responsible for funding acquisition. S.M.G. was responsible for project administration, validation, visualisation and writing – original draft. All authors were responsible for review and editing of the draft. All authors have agreed on the final version of this manuscript.

## Disclosure

Submission with statistics: The authors have checked to make sure that our submission conforms as applicable to the Journal's statistical guidelines. The statistics reported in this paper span descriptive statistics, summaries and basic relationship testing between variables. The second author, MP, has a background in data analytics. The authors affirm that the methods used in the data analyses are suitably applied to their data within their study design and context, and the statistical findings have been implemented and interpreted correctly. The authors agree to take responsibility for ensuring that the choice of statistical approach is appropriate and is conducted and interpreted correctly as a condition to submit to the Journal.

## Conflicts of Interest

The corresponding author, S.M.G., has a prior perceived familial conflict of interest in regard to Medi‐Map Ltd., with two family members occupying senior roles at this company up until December 2023. The data request to this company was made in March 2024 and managed by the Chief Technical Officer overseeing Medi‐Map as part of Z Software, who had no prior personal or professional relationship with the corresponding author (S.M.G.). The other authors list on this paper declare that they have no known competing financial interests or personal relationships that could have appeared to influence the work reported in this paper.

## Supporting information


Appendix S1

## Data Availability

Research data are not shared.
